# Development of the social frailty scale for older adults under pandemic-related social restrictions

**DOI:** 10.3389/fragi.2026.1763012

**Published:** 2026-06-24

**Authors:** Pei-Hsin Fang, Ray-E. Chang, Robert C. Myrtle, Ying-Hui Hou, Ya-Mei Chen

**Affiliations:** 1 Center for Physical Education and Sports, Southern Taiwan University of Science and Technology, Tainan City, Taiwan; 2 Institute of Health Policy and Management, National Taiwan University, Taipei, Taiwan; 3 Center for Operations and Strategy, Dajia Lee’s General Hospital, Taichung, Taiwan; 4 Sol Price School of Public Policy, University of Southern California, Los Angeles, CA, United States; 5 SMS InfoCom Corporation, Grapevine, TX, United States

**Keywords:** aging, community-dwelling older adults, frailty, scale development, social domain

## Abstract

**Introduction:**

Social frailty, a key dimension of the multidimensional assessment of frailty, has been linked to poor health outcomes and poorer quality of life in older adults. However, definitions and measurements of social frailty vary and most do not provide assessments of reliability or validity. This study sought to identify the core components associated with social frailty and to create a reliable, valid social frailty scale.

**Methods:**

This study adopts a psychometric approach to develop a social frailty scale for use in a community context under pandemic-related social restrictions. Systematic searching, content analysis, and a modified Delphi method were applied to develop a draft of a social frailty scale. Item-analysis, explorative factor analysis, confirmatory factor analysis, and reliability analysis were used to test scale properties. The subjects were older adults above 65 years old who live in Taiwan, and 446 people in total were interviewed to collect data.

**Results:**

The results indicated that five factors (two types of social support, social participation, social networks, and loneliness) and 16 items formed a social frailty scale with appropriate reliability and validity. This study advances our understanding of core components of social frailty.

**Discussion:**

The social frailty scale integrates several social frailty instruments to detect social frailty in community context.

## Introduction

Previous studies have focused on frailty from a physical perspective, indicated that frailty may be a physiologic precursor to disability and be related to a high risk of adverse health outcomes (Fried et al., 2001). Gobbens et al. offers a more inclusive perspective, pointing out that “frailty is a dynamic state affecting an individual who experiences losses in one or more domains of human functioning (physical, psychological, social), which is caused by the influence of a range of variables and increases the risk of adverse outcomes” (Gobbens et al., 2010a).

This concept of frailty, to include the physical, psychological, cognitive, and social domains, stimulated a proliferation of research focusing on the multi-domain aspects of frailty (Van Oostrom et al., 2017). One of the areas of interest associated with multi-domain frailty is social frailty. Evidence shows that social frailty is related to poor quality of life (Zhang et al., 2019), increased the risk of physical frailty (Makizako et al., 2018), disability (Makizako et al., 2015; Teo et al., 2017), and cognitive impairment (Tsutsumimoto et al., 2017).

Although the social domain of frailty has received considerable research attention, there has not been a widely accepted definition and instrument for measuring social frailty. Bunt et al. started with the basic proposition that social frailty could be considered as a lack of resources to fulfill one’s basic social needs. They define social frailty as “a continuum of being at risk of losing, or having lost, resources that are important for fulfilling one or more basic social needs during the life span” (p.323) (Bunt et al., 2017). Subsequent studies followed this definition for discussing social frailty (Teo et al., 2017; Bessa et al., 2018).

Other scholars have conceptualized the social domain of frailty through the perspective of social ecology (Andrew and Keefe, 2014). They note that many social factors have the potential to influence an older person’s health. Since social contexts are inherently dynamic it is important to recognize these changes can evolve over the short and long term. As such changes in the social factors (such as social support, social engagement, social networks, and socioeconomic status) may be seen as contributing to and/or mitigating the impact on this social domain. While various explanations have been offered to explain how these social factors may affect health outcomes, most studies identify several social factors as important elements contributing to social frailty. Specifically, social networks (Burholt et al., 2017; Hughes et al., 2008; Martin et al., 2001), social support (Hughes et al., 2008; Isherwood, 2023), and social engagement or social participation (Martin et al., 2001; Isherwood, 2023) have been identified as having an impact on health outcomes.

Various instruments have been developed to assess how these relationships affect social frailty. However, the diversity in these tools, specifically regarding their components and question structures, suggests a need for a more holistic understanding. This complexity is illustrated by Bessa et al. (2018), whose systematic review found 11 different factors encompassing the social dimension of frailty. Although most research has found that changes in the physical, psychological and cognitive domains have been associated with different components of social frailty, unresolved is the relative importance of these different components in assessing social frailty.

Another challenge in assessing social frailty is that of including questions from assessments that focus on different concerns associated with social frailty. As summarized in Supplementary Table S1 these issues were associated with social capital assessments (Ehsan et al., 2019; Moore and Kawachi, 2017), social network considerations (Smith and Christakis, 2008), social support mechanisms (Langford et al., 1997), and aspects of social participation (Ang, 2018; Dawson-Townsend, 2019). This divergence is best illustrated in the differing descriptions between social capital and social frailty (Supplementary Table S1).

In addition, many of the social components and items included in the different assessments for measuring social frailty have not been validated for that purpose. More importantly, only a few studies examining multi-dimensional frailty have used a psychometric approach to demonstrate the assessment tools’ reliability and validity (Sutton et al., 2016) or have conducted cross-national scale validation, such as for the Tilburg Frailty Indicator (TFI) (Gobbens, et al., 2010b) or the Groningen Frailty Indicator (GFI) (Schuurmans, et al., 2004). However, these two instruments were designed to provide a comprehensive assessment of frailty. As such, the social frailty domains and the questions assessing those areas were limited. Not to diminish the value of these comprehensive assessments of frailty, each relies on three questions to assess one or more domains associated with social frailty. Yet, as Bunt et al. (2017) point out, social frailty is one of the most complex subdomains within the multidimensional construct of frailty in older adults. Rojas-Avila et al. (2025) point out that social frailty has been associated with various adverse health conditions and as such highlight the need for a deeper understanding of its components and mechanisms. Moreover, some social factors included in the different assessments of social frailty are not necessarily adverse for frailty or other health outcomes. For example, living alone is frequently used to assess social frailty, yet several longitudinal studies indicated that it did not predict frailty (Bessa et al., 2018; Bessa et al., 2021). And, as noted by Besse et al. (2018) some social influences cover a diverse set of domains, such as social support, which in turn include dimensions pertaining to emotional, instrumental, informational and appraisal support issues (Mohd et al., 2019).

As noted above, assessing social frailty facilitates the design of preventive interventions to improve the health of older adults. However, unless reconciled, these differences raise questions about the adequacy of current assessments of social frailty. This underscores the academic importance of the present study, which examines the components of social frailty to enhance our ability to assess this concern. To address these issues, this study conducted a systematic literature review to retrieve existing assessments for measuring social factors of frailty or social frailty, and to determine the core factors—specifically those most frequently utilized—and their corresponding items within these scales. Subsequently, a psychometric approach was used to develop a social frailty scale with appropriate levels of measurement reliability and validity.

## Methods

### Study design

**FIGURE 1:**
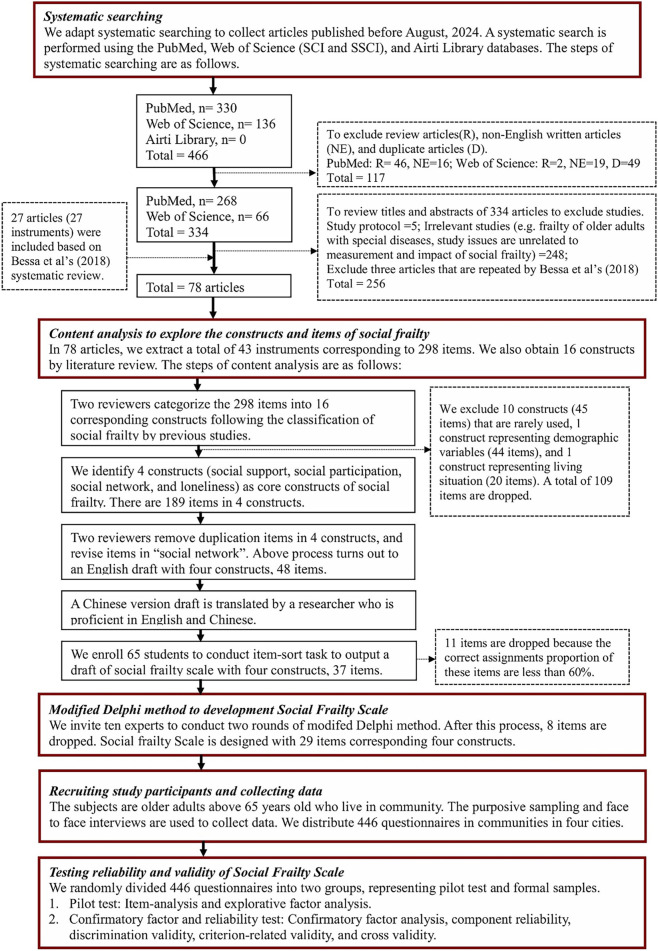
Flow diagram of study.

This study uses an integrated set of methods outlined in Figure 1.

### Systematic searching

The results from Bessa et al.’s (2018) systematic review provided a beginning point. We added to their work by following a systematic search process using PubMed, Web of Science (SCI and SSCI), and Airti Library databases encompassing all years up to 1 August 2024. The searching strategy was noted in SI Appendix. Once the studies were identified, two independent reviewers reviewed the titles and abstracts to exclude review, duplicate, non-English, and irrelevant articles. Full text versions were retrieved and reviewed by the same independent reviewers to identify the research instruments used in the selected works. In the rare instance where the reviewers disagreed on the inclusion and exclusion of articles, a third reviewer was used to resolve the disagreement.

### Content analysis

Since surveys differed in the questions and the categories used to measure social frailty, we conducted a process of item-sort to reconcile these differences. Sixty-five undergraduate and graduate students were recruited to assist in the item-sort process. Verbal instructions and written directions were provided and the students were asked to review a list of randomly listed questions and identify the category they believed each question belong to. The results of this process were analyzed using a proportion-of-substantive-agreement index proposed by Anderson and Gerbing (1991). Those items which were found to have a substantive-agreement index of 60% or more were retained (Huang et al., 2012).

### Modified delphi method

Ten experts drawn from the fields of public health, long term care, gerontology, and psychology formed the Delphi panel. Using the questions retained by the item-sort process, the panel participants reviewed each item and using a 5-point Likert Scale, indicated the extent of their agreement with the significance and/or appropriateness of each. In order to assess stability and the degree of agreement of an item, the criteria used to retain an item was a mean greater than 4.00, a standard deviation less than 1, and a quartile deviation is less than 1 (Faherty, 1979).

### The processes of scale testing

After developing a draft of the social frailty scale, we sought to examine the scale’s reliability and validity. In process of item-analysis, 6 criteria including missing data, descriptive statistics testing, comparisons of extreme groups and homogeneity testing were applied (Chiou, 2018). Items that did not meet three of these criteria were excluded. Exploratory factor analysis was used to identify the factor structure of social frailty scale. Confirmatory factor analysis was applied to test the construct validity of latent variables by structural equation modeling. We used physical frailty, social frailty (the Questionnaire to define Social Frailty Status, QSFS), Instrumental Activities of Daily Living (IADL), and quality of life for evaluating criterion-related validity. We also examined the known-group validity, multi-group analysis, internal consistency reliability, and the split-half reliability. The result turned out to be statistically significant at ɑ = 0.05 level.

### Participants and process

The subjects were older adults 65 years or older who live in Taipei City, New Taipei City, Miaoli County, and Tainan city. Purposive sampling and face to face interviews were used to collect data from August 2020 to February 2021. Older adults who live with dementia or who could not complete the tests of physical frailty were excluded. We recruited 446 participants aged from 65 to 100 years. We randomly selected about 50% of the sample (n = 227) to conduct a pilot test (including items-analysis and explorative factor analysis). We then conducted a confirmatory factor analysis and reliability analysis using the other sample (n = 219).

### Measurements

Four well established research assessments were used for criterion-related validity testing. The Fried’s frailty phenotype was used to evaluate physical frailty (Fried et al., 2001). The “Lawton-Brody’s Instrumental Activities of Daily Living” was used to evaluate participants’ important domains of functioning (Lawton and Brody, 1969). The “WHOQOL-OLD-Taiwan version” (Yao et al., 2017) was used to measure quality of life. Finally, the “Questionnaire to Define Social Frailty Status (QSFS)” (Makizako et al., 2015) was used to assess social frailty.

## Results

### Systematic review

The Systematic Search Process identified 78 articles that met the selection criteria. A total of 43 instruments corresponding to 298 questions and 16 corresponding constructs were obtained from these articles (Bessa et al., 2018; Andrew and Keefe, 2014; Andrew et al., 2008) (Supplementary Tables S2, S3).

Ten constructs that were used by 1 or 2 scales, one construct representing demographic variables and one construct representing living alone were excluded. A previous study reported living alone is not absolute disadvantage in terms of health status (Michael et al., 2001). In addition, Molina-Mula, et al. (2020) indicated that older adults that despite their loneliness, may want to continue to live alone.

Even though loneliness is not considered to be a social resource, items related to loneliness are included in commonly used scales (such as TFI and GFI). In order to confirm the constructs of social frailty, we included loneliness in the analysis process. At the end of this process four constructs: social support, social participation, social network, and loneliness, emerged as the most frequently used social components with 189 items.

We then removed duplicative items in the four constructs, and revised several items in the “social network” construct. This resulted in a questionnaire draft with four constructs and 48 questions that became the basis for the item-sort analysis. When these questions were evaluated by the item-sort process, 37 of the 48 questions were found to be associated with the 4 constructs. Eleven items were dropped because they did not meet the proportion-of-substantive-agreement index requirement of 60% or more suggested by Huang, et al. (2012).

### Modified delphi method

In the first round, 7 of the 37 items were dropped because of a low degree of support for inclusion by the panel. Fifteen of the items needed minor revisions. The second round found that all items in that round met the consensus criteria. One item “Do you go out at least once a week?” was deleted because experts suggested that the meaning of this item was unclear. At the conclusion of this process 29 items were identified as appropriate measures of social frailty.

### Items-analysis

The pilot test participants’ responses (227) were used in the item and factor analysis findings reported here. Following the process outlined by Chiou (2018), two of the 29 question items did not meet the retention requirements (Supplementary Table S4).

### Explorative factor analysis

As reported in Table 1, seven factors and 18 items formed a measure that accounted for 78.01% of the total variance. The first and third factors contain items that seem to reflect “instrumental social support” (item 7–9) and “emotional social support” (item 1–3). These two factors, emotional social support and instrumental social support are similar to social support measures identified in other studies (Mohd et al., 2019). The second factor represents “social participation” (item 12, 13, and 15). The fourth factor represents loneliness (items 24, 25, and 27). The fifth to seven factors represent social network interactions with family, friends, and neighbors. However, as the items shared the same underlying meaning and differed only in terms of the target of interaction, they were combined and collectively named “social networks”.

**TABLE 1:** Analysis of explorative factor analysis (N = 227).

Items	Factors						
	1	2	3	4	5	6	7
1. Is there someone caring about you?	0.315	0.054	0.710	0.173	0.080	−0.032	0.031
2. Do you have someone to confide in?	0.114	0.127	0.826	−0.023	0.042	0.123	0.025
3. Is there someone comforting you?	0.223	0.124	0.842	0.019	−0.020	0.111	0.161
7. Do you have someone you could ask for help if you were in case of illness or emergency (e.g., sudden fall)?	0.874	0.085	0.233	0.081	0.064	0.047	0.038
8. Do you have someone you could ask for help if you were in a crisis?	0.830	0.152	0.274	0.082	0.074	−0.007	0.034
9. Do you have someone you could ask for help if you need help with transportation?	0.875	0.020	0.113	0.050	0.003	0.121	0.119
12. How often do you participate in social activities?	0.077	0.828	0.171	0.054	0.176	0.168	0.040
13. How often do you participate in community activities?	0.113	0.829	0.048	−0.015	0.122	0.150	−0.008
15. How often do you participate in group activities?	0.046	0.868	0.094	0.071	0.055	0.153	0.030
18. How often do you contact with family through line/FB, email, etc.,?	0.029	0.072	0.078	−0.042	0.162	0.132	0.858
19. How often do you meet your family?	0.132	−0.023	0.092	0.085	0.101	−0.030	0.864
20. How often do you contact with friends through line/FB, email, etc.,?	0.088	0.227	0.173	0.076	0.155	0.857	0.131
21. How often do you meet your friends ?	0.065	0.258	0.035	0.083	0.136	0.877	−0.010
22. How often do you contact with neighbors through line/FB, email, etc.,?	0.020	0.144	0.054	0.084	0.890	0.164	0.141
23. How often do you meet your neighbors?	0.099	0.179	0.030	0.007	0.891	0.107	0.144
24. Do you feel lonely?	0.017	−0.008	0.055	0.791	0.081	0.116	0.098
25. Do you feel rejected?	0.051	0.093	0.092	0.775	0.092	−0.006	−0.084
27. Do you feel sense of emptiness?	0.118	0.011	−0.024	0.839	−0.089	0.031	0.036
Explained variance %	13.663	13.201	11.893	11.185	9.745	9.407	8.921
The total variance of scale %	78.014						

Factors selected had eigenvalues greater than 1. We deleted items which cross loaded or whose loading was <0.55 [28].

### Confirmatory factor analysis

**FIGURE 2:**
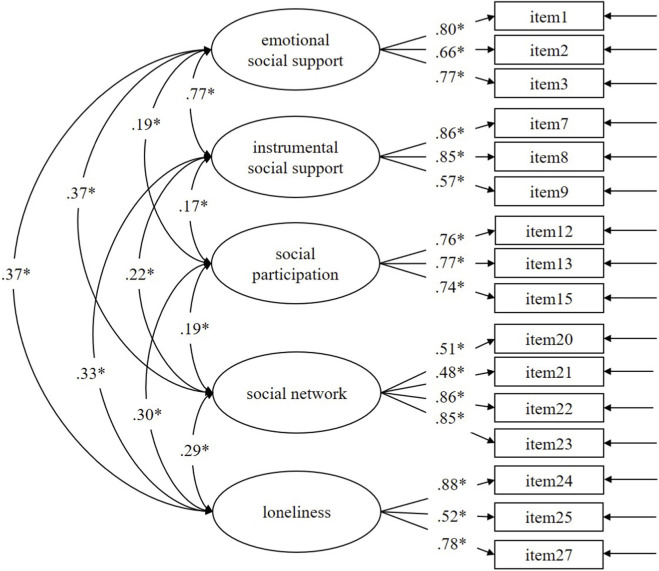
First-order oblique model of confirmatory factor analysis.

Using the responses from the total sample size (219), we tested the model fit of first-order oblique model and second-order model. The first-order oblique model yielded better fit indices than the second-order model; however, the values remained below the recommended thresholds. Based on the model modification indices, two items pertaining to the family social network dimension were excluded due to low standardized factor loadings and high residual values; the revised model subsequently demonstrated adequate fit. Finaly, the model was tested using a different pilot sample (227) and the full sample (446), both of which yielded good model fit (Table 2; Figure 2). The component reliability of instrumental social support, emotional social support, social participation, social network (friends and neighbors), and loneliness were from 0.78 to 0.81 (Supplementary Table S5). All values are greater than 0.60 (Wu, 2009) which indicates that the scale for social frailty had high reliability. The average variance extraction (AVE) of instrumental social support, emotional social support, social participation, and loneliness were greater than 0.50, however that of the social network was 0.49 which is slightly less than the 0.5 criterion suggested by Wu (2009).

**TABLE 2:** Analysis of competing models (N = 219).

Model	*χ* ^2^/df	GFI	AGFI	RMSEA	NFI	CFI	IFI	TLI
Criterion of model fit	<3	>0.9	>0.9	<0.08	>0.9	>0.9	>0.9	>0.9
First-order oblique model	3.398	0.818	0.752	0.105	0.759	0.814	0.817	0.772
Second-order model	3.423	0.811	0.751	0.105	0.748	0.805	0.807	0.77
Modified first-order oblique model	1.935	0.908	0.863	0.065	0.886	0.94	0.942	0.922
Modified first-order oblique model (sample size = 446)	2.253	0.945	0.919	0.053	0.931	0.961	0.962	0.949
Modified first-order oblique model (sample size = 227)	1.651	0.925	0.890	0.054	0.911	0.962	0.963	0.951

### Model testing by known-group validity and multi-group analysis

We test the known-group validity using one-way ANOVA. The result showed that there was significant difference in total score of social frailty (*F* = 11.402, *p* < 0.05) between participants with physical frailty (N = 46), participants with pre-physical frailty (N = 205), and robust participants (N = 194). Post hoc comparisons further shows that the total score of social frailty of participants with physical frailty is higher than prefrail and robust participants.

This study conducted a multi-group analysis across genders (n = 100 for male and n = 346 for female) to test the model, employing a procedure that progressed from least to most restrictive. The results indicated that the unconstrained model exhibited a good fit (χ^2^/df = 1.972, RMSEA = 0.047, CFI = 0.940, TLI = 0.922, IFI = 0.942, GFI = 0.911). Regarding the comparison of competing models, the results for model measurement weights were χ^2^ = 10.135, df = 11, p = 0.473; for model structural covariances, χ^2^ = 26.483, df = 26, p = 0.697; and for model measurement residuals, χ^2^ = 64.964, df = 44, p = 0.022. Invariance was supported across all levels except for the most stringent measurement residuals model. The other multi-group analysis across education levels (n = 86 for no formal education, n = 168 for primary education, and n = 192 for secondary education and higher) was conducted to test the model. The results indicated that the unconstrained model exhibited an acceptable fit (χ^2^/df = 2.419, RMSEA = 0.057, CFI = 0.861, TLI = 0.843, IFI = 0.862, GFI = 0.811). Regarding the comparison of competing models, the results for model measurement weights were χ^2^ = 10.949, df = 11, p = 0.448; for model structural covariances, χ^2^ = 43.535, df = 26, p = 0.017. Measurement invariance was supported by the model measurement weights.

### Criterion-related validity

The criterion-related validity analysis found that the total social frailty score had a statistically significant correlation with QSFS (*r* = 0.36), physical frailty (*r* = 0.19), IADL (*r* = −0.16), and quality of life (*r* = −0.48) (Supplementary Table S6). Supplementary Tables S7, S8, the hierarchical regression analysis indicated that social frailty scale (SF-16) performed better and represented an important improvement in the assessment of quality of life over the QSFS. Observing the △R^2^ of the models, the △R^2^ of model 1 (QSFS) was 7.2%. Adding the SF-16 (Model 2) increased the △R^2^ by 16.9% (R^2^ = 0.241). When we reversed the entry of the SF-16 and QSFS measures the R^2^ was 0.230. Adding the QSFS measure the R^2^ was increased by 0.011 (R^2^ = 0.241).

### Reliability analysis

The Cronbach’s α of emotional social support, instrumental social support, social participation, social network and loneliness were 0.786, 0.842, 0.823, 0.779, and 0.751, respectively. The overall internal consistency of the scale attains 0.835. Furthermore, the split-half reliability is estimated by Spearman-Brown correlation. The result shows that the correlation of two parts of scale is 0.618. Our scale of social frailty demonstrated satisfactory internal consistency.

## Discussion

Through a systematic literature review and synthesis of prior research, this study extends the conceptual definition of social frailty proposed by Bunt et al. (2017)—defined as “a continuum of being at risk of losing, or having lost, resources that are important for fulfilling one or more basic social needs”—by identifying and incorporating social support, social participation, social networks, and loneliness as core dimensions of the construct. This study employed a psychometric approach to develop the scale, ensuring appropriate reliability and validity through rigorous testing, including item analysis, exploratory factor analysis (EFA), confirmatory factor analysis (CFA), and reliability analysis. Compared to other existing instruments, this scale integrates core factors such as social support (both emotional and instrumental), social participation, social networks (friends and neighbors), and loneliness. Furthermore, this scale adopts a 4-point Likert scale for response options instead of the “yes/no” dichotomy used by most existing instruments (such as the QSFS, TFI, Social Vulnerability Index, and Frailty Index), enabling a more nuanced reflection of the social frailty status of older adults. In terms of criterion-related validity, it is worth noting that the significant moderate correlation between social frailty and quality of life presents appropriate criterion-related validity. Compared with the QSFS, the Social Frailty Scale provided a better assessment of the quality of life of older adults. However, the Social Frailty Scale was only weakly correlated with the Physical Frailty and the IADL measures. Since physical frailty and disability usually occur in a relative older age, that might be a factor influencing these results. It is also possible that the variability in the relationship between social frailty and physical frailty may have an impact on the statistical results. According to Makizako et al. (2018), social frailty may precede and lead to physical frailty. By contrast, Nagai et al. (2020) found that physical frailty symptoms such as slow gait speed and muscle weakness predict the development of social frailty.

### Social support

Social support is the most common concept used to measure social frailty. However, items representing social support vary and cover different forms. The EFA result found that social support involved two different types of activities, namely, instrumental social support and emotional social support. Our findings were consistent with studies that use instrumental support as well as emotional support as measurement variables assessing the relationship between older adults’ health (Luo et al., 2021; Saito et al., 2005). Interestingly, items representing professional advice (e.g., medical, law, finance, and social welfare) seeking and advice asking from family (or relatives/friends) were dropped in EFA process. This suggests that advice seeking may not be as important as emotional and instrumental social support in assessing social frailty. “Elder respect” is a general traditional value in Chinese society. Furthermore, older adults often enjoyed authority in their families because of traditional culture (Lu et al., 2002). Therefore, older adults may be less likely to ask for advice from relatives, friends or others when they encounter problems. It would be worthwhile to verify this result in different culture contexts.

### Social network

The contact frequency, network size and diversity of social networks have been used in studies in relation to health outcomes (Sharifian et al., 2022). There are significant between-study differences with respect to definitions and measurements of social networks. Most studies used one or two items to represent the social network. Since social networks can be more complex, we integrated the concepts of contact frequency (always, frequently, a little, not at all), contact type (meet and contact), and diversity (families, friends and neighbors) to create 6 items to represent the social network construct. Following the confirmatory factor analysis, items related to family within the social network scale were removed. It’s predictable that the social networks of older adults will shift throughout the life course along with life transitions and events. Having a more diverse social network may lead to more opportunities for social participation (Ali et al., 2018). Also, the diversity of social network can be more important than the size of social network because of the need for changes in the type support associated with the aging process.

The findings of this study prompt a reconsideration of the heavy emphasis placed on family structure and support in Chinese societies, which may lead to overlooking the potential of developing friend networks as an alternative source of social support. The results suggest that the social networks of neighbors and friends might be more important than family in a social frailty context. This may be a reflection of a changing family structure in Chinese context as children grow up and establish their careers and families away from their parents’ resident areas. Also, the increasing numbers of older adults who are without children or a spouse in later life make them therefore more likely have greater reliance on friends (Mair, 2019). Research shows that abundant friends’ networks can reduce the risk of adverse outcomes of health (Litwin, 2011). Interestingly, a Chinese study found that older adults were more likely to join a network with friends than a family one as one aged (Li et al., 2018).

### Social participation

The construct of social participation in Social Frailty Scale has strengths for measurement practice. Compared to the items in other instruments that often use a “yes/no” format, the responses were based on the extent of participation. Instead of listing a series of possible social activities in one question, this survey specifies a number activities that have been used to assess social participation. The social participation component of this survey also highlighted the importance of social networks and social resources for older adults. In the Taiwanese context, the government invests considerable resources to organize group activities and community activities for older adults. Aside from support from government resources for older adults, building social networks as well as obtaining formal and informal social support, were the reasons for putting importance on social participation. Some studies targeting friend’s network support our discussion. Sharifian et al. (2020) discovered that more frequent contact with friends, but not family, was associated with greater concurrent engagement in physical and cognitive activities.

### Loneliness

Many instruments of social frailty have items related to loneliness because loneliness has been regarded as an important risk for older adults’ health. Loneliness is a negative feeling related to loss and disappointment, and all negative loneliness feelings pointing to a lack of connectedness with people and life (Van Tiburg, 2021). Although sometimes considered synonymous with living alone, loneliness and living alone are related but distinct categories. Loneliness is more closely associated with deficits in the perceived quality of one’s social interactions (Ong et al., 2016). Consequently, including loneliness might allow us to move beyond the limitations of objective living status (such as living alone) and instead evaluate deficits in the perceived quality of social interactions or relational resources among older adults.

Previous studies illustrated that older adults who lack of social support (Hu et al., 2021), have a limited social network (Victor and Bowling, 2012), with less social engagement (Park, et al., 2020), and were more likely to feel lonely. Although loneliness has been linked to frailty, research suggests that improving social relationships may reduce experienced levels of loneliness (Victor and Bowling, 2012). We included several loneliness items in the development of our survey and our findings offer support for previous studies. We conclude that loneliness is an appropriate consideration in the assessment of social frailty.

### Construct deleted

We dropped the “living alone” construct during the content analysis because previous research found that receiving emotional and instrumental social support from the community contributes to the health benefits of older adults who live alone (Saito et al., 2005). Studies also suggested that the effects of social network may exceed the impact of living alone (Sakurai et al., 2019). Research has illustrated that older adults who lack of social support (Hu et al., 2021), have a limited social network (Victor and Bowling, 2012), with less social engagement (Park et al., 2020), were more likely to feel lonely.

## Limitations

This study had several limitations. First, the purposive sampling applied in this study may have limited the generalizability of the findings. Second, during the scale validation process, some of the excluded items may reflect the cultural specificities of the Chinese context. We suggest that future research should validate this scale in diverse cultural settings, particularly by re-examining whether the deleted ‘advice-seeking’ items demonstrate stronger psychometric properties in other cultural populations. Third, test–retest reliability was not examined to evaluate the stability of the responses. Fourth, regarding criterion-related validity, this study primarily focused on social frailty, physical frailty, and quality of life, without incorporating multidimensional frailty scales into the analysis. Future research could include widely used multidimensional assessment tools, such as the Tilburg Frailty Indicator (TFI) and the Groningen Frailty Indicator (GFI), for further comparison. Fifth, this study was conducted during the COVID-19 pandemic, a period when pandemic-related restrictions may have dampened individuals’ willingness to engage in social activities. Furthermore, while the sample did include participants categorized as frail or pre-frail, those who volunteered were likely more active and healthier than non-participants in the same community. Consequently, this potential selection bias may have compromised the sample’s representativeness, leading to an underestimation of the actual prevalence of social and physical frailty in the broader population. Future studies could re-validate the scale in a post-pandemic context where social restrictions are no longer in place. This would help determine if the factor structure and item responses remain stable. Finally, national culture differences and government actions might may have an influence on assessments of social frailty and could be an important consideration in future research. Hofstede’s (1984) depiction of the Chinese culture as placing greater value on collectivism compared to individualism may also have had an influence on the lack of support for the living alone construct.

## Data Availability

The raw data supporting the conclusions of this article will be made available by the authors, without undue reservation.
