# Supplementary material for: Development of the social frailty scale for older adults under pandemic-related social restrictions
Source: Front Aging. 2026 Jun 24;7:1763012. doi: 10.3389/fragi.2026.1763012 (PMC13341860; doi:10.3389/fragi.2026.1763012)
Supplement: Supplementary file 1 [file DataSheet1.pdf]

Supplementary Material

Searching strategy

("frailty"[MeSH Terms] OR "frailty"[Title/Abstract]) AND (("aged"[MeSH Terms] OR "aged"[Title/Abstract] OR "elderly"[Title/Abstract] OR "older adult"[Title/Abstract])) AND (("social vulnerability "[Title/Abstract] OR "social frailty"[Title/Abstract] OR "social dimension"[Title/Abstract] OR "social function"[Title/Abstract] OR "loneliness"[Title/Abstract] OR "social support"[Title/Abstract] OR "social network"[Title/Abstract] OR "social engagement"[Title/Abstract] OR "social participate"[Title/Abstract] OR "social relation"[Title/Abstract] OR "social relationship"[Title/Abstract])) NOT "abuse"[Title/Abstract]).

Note: We exclude studies that include “abuse” as a keyword because we found that when it was included it returned research that was not related to the focus of this study.

Table S1: Description of social academic terms

Academic noun	Concept or definition
Social frailty	Bunt et al (2017) defined social frailty as “a continuum of being at risk of losing, or having lost, resources that are important for fulfilling one or more basic social needs during the life span.”
Social capital	Social capital (SC) refers to features of social organization, such as networks, norms, and trust, that facilitate coordination and cooperation for mutual benefit. SC enhances the benefits of investment in physical and human capital (Putnam, 1994). Contemporary SC and health research considers social cohesion and social network approaches to health. The cohesion approach tend to emphasis the cognitive or structural side of social capital through questions about trust in others, perceptions of social belonging and integration, and levels of civic or social participation. The network approach tend to rely on formal social network analysis (SNA) methods to measure social resources and networks, while highlighting inequalities in access to social resources. SC has been conceptually defined and measured at the individual and community (group) levels (Dawson-Townsend, 2019; Ehsan et al., 2019; Moore & Kawachi, 2017).
Social networks	According to Smith and Christakis’ work (2008), studies operationalized social networks as an individual-level measure of the number of social contacts a person has (structural support, or its quantitative aspect) or how helpful they are, as subjectively reported by the person (functional support, or its qualitative aspect). Social network studies characterize the web of social relations around an individual, including, most importantly, who the contacts are and the nature of the ties that connect them. For example, one might look at particular characteristics of those who make up a person’s support network or the type of links (e.g., close/distant, friend/relative) that connect them.
Social support	According to the work of Langford et al. (1997), social support refers to the various sources of help and resources obtained through social relationships with family, friends, and other care providers. Types of social support include emotional (including the presence of a close confidante), instrumental (help with activities of daily living, provided through labor or financial support), appraisal (help with decision making), and informational (provision of information or advice). Social network, social embeddedness, and social climate were identified as antecedents of social support.
Social participation	Most scholars have argued that social participation consists of two main components – formal and informal participation. Formal social participation entails involvement in formal groups and organizations in the community, while informal social participation refers to the level of contact and time spent with family and friends (Ang, 2018).

1. S. Bunt, N. Steverink, J. Olthof, C. Van Der Schans, J. Hobbelen, Social frailty in older adults: a scoping review. European journal of ageing 14, 323-334 (2017).

2. R. D. Putnam, Social capital and public affairs. Bulletin of the American Academy of Arts and Sciences, 5-19 (1994).

3. K. Dawson-Townsend, Social participation patterns and their associations with health and well-being for older adults. SSM-population health 8, 100424 (2019).

4. A. Ehsan, H. S. Klaas, A. Bastianen, D. Spini, Social capital and health: A systematic review of

systematic reviews. SSM-population health 8, 100425 (2019).

5. S. Moore, I. Kawachi, Twenty years of social capital and health research: a glossary. J Epidemiol Community Health 71, 513-517 (2017).

6. K. P. Smith, N. A. Christakis, Social networks and health. Annu. Rev. Sociol 34, 405-429 (2008).

7. C. P. H. Langford, J. Bowsher, J. P. Maloney, P. P. Lillis, Social support: a conceptual analysis. Journal of advanced nursing 25, 95-100 (1997).

8. S. Ang, Social participation and health over the adult life course: Does the association strengthen with age? Social Science & Medicine 206, 51-59 (2018).

Table S2: Description of instruments

No	Authors /year	Instrument	Description	Numbers of items	Numbers of social items	Items
1	Barber, et al. (1980)	Postal Screening Questionnaire, PSQ	PSQ is a multidimensional screening tool which identifies individual who may be at risk of dependency, functional status, psychosocial functioning, neurosensory deficits, health, social support and hospital admissions. Each of the nine questions representing an aspect of health and well-being and is so phrased that the answer 'Yes' to any one would be considered an indicator of an at risk state, the individual thus being in need of assessment.	9	2	1. Do you live on your own?<br>2. Are you without a relative you could call on for help?
2.1<br>2.2	Cacciatore, et al. (2005); Lachs, et al.(1990)	Frailty Staging System, FSS	The FSS is an index of severity of functional impairment which is developed by Lachs, et al.(1990). The FSS is consisted of disability, mobility, cognitive function, visual function, hearing function, urinary continence and social support. For each domain the score is equal to 0 when the function is preserved and it is equal to 1 when the function is lost.	7	1	1. Who will able to help you in case of illness or emergency?
3	Hébert, et al. (1996)	Sherbrooke Postal Questionnaire, SPQ	The SPQ is a tool for the screening of community dwelling elderly at risk for functional decline. The original version is a 21-item questionnaire. Each question is formulated in a very simple style to be answered yes or no, except for the marital status question. After questionnaire testing, a short index including age, sex, and six questions on disability is considered that it was useful in identifying disabled subjects.	8	5	1. Do you need someone to help you on a regular basis<br>2. In case of need, can you count on someone close to you<br>3. In general, do you have any health problems that require you to limit your activities?<br>4. Age (>= 85)<br>5. Sex (male)
4	Saliba, et al. (2001)	The Vulnerable Elders Survey-13, VES-13	The VES-13 is composed of 13 items relating age, self-rated health, six physical function limitations, and five IADL/ADL items.	13	1	1. Age
5	Schuurmans, et al. (2004)	The Groningen Frailty Indicator, GFI	The GFI is composed of 15 items across 4 domains: physical (mobility functions, multiple health problems, physical fatigue, vision, hearing), cognitive (cognitive functioning), social (emotional isolation), and	15	3	1. Do you sometimes experience emptiness around yourself?<br>2. Do you sometimes miss people around yourself?<br>3. Do you sometimes feel abandoned?

			psychological (depressed mood and feelings of anxiety).			
6	Rolfson, et al. (2006)	The Edmonton Frail Scale, EFS	The EFS is composed of 11 items across 9 domains (cognition, general health status, functional independence, social support, medication use, nutrition, mood, continence, and functional performance). The maximum score of the EFS is 17 and represents the highest level of frailty.	11	1	1. When you need help, can you count on someone who is willing and able to meet your needs
7	Raîche, et al. (2008)	The Prisma-7	The Prisma-7 consists of seven items corresponding to socio-demographic variables, autonomy and support. It is coded by yes/no answer, and three or more positive answers identify individuals as at risk of disability.	7	5	1. Are you older than 85 years?<br>2. Are you male?<br>3. In general, do you have any health problems that require you to limit your activities?<br>4. Do you need someone to help you on a regular basis?<br>5. If you need help, can you count on someone close to you?
8	Gobbens, et al. (2010)	Tilburg Frailty Indicator, TFI	The TFI is composed of two parts. Part A includes questions on the determinants of frailty, divided by ten questions on sociodemographic data, lifestyle, morbidity, life events and environment. Part B assesses frailty through 15 items divided physical, psychological and social dimensions.	25	8	1. Part A.Which sex are you?<br>2. Part A.What is your age?<br>3. Part A.What is your marital status?<br>4. Part A.What is the highest level of education you have completed?<br>5. Part A.Which category indicates your net monthly household income?<br>6. Part B.Do you live alone?<br>7. Part B.Do you sometimes miss having people around you?<br>8. Part B.Do you receive enough support from other people?
9	De Witte, et al. (2013)	Comprehensive Frailty Assessment Instrument, CFAI	The CFAI captures frailty on physical, psychological (mood and emotion), social (social loneliness and social support network) and environmental domains. A total of 23 items is included in CFAI.	23	4	1. Social loneliness: There are plenty of people I can lean on when I have problems.<br>2. Social loneliness: There are many people I can trust completely.<br>3. Social loneliness: There are enough people I feel close to.<br>4. Social support network 1; partner, son and daughter-in-law; Social Network 2; Daughter, son-in-law and grandchildren; Social Network 3; brother or sister (-in-law), family, neighbors and friends.
10	van Kempen, et al. (2013)	The Easycare Two-Step	The Easycare-TOS is divided into two steps. In first step, the GP reviews the patient record and answers 14 questions about the	25	6	1. First Step. Loneliness, patient has: No loneliness; Had complaints of loneliness in the past 12 months; Unknown.

		Older Persons Screening, Easycare-TOS	functioning of the patient in somatic, psychological, and social domains to decide whether the patient is frail. The patients who are judged as ‘unclear’ or ‘frail’ in the first step are eligible for the second step. In the second step, additional information is collected through a structured assessment by a primary care nurse. The scale using in second step includes background variables and 11 questions relating with multimorbidity, cognitive problems, memory test, mobility and falling, looking after yourself, Seeing/ hearing/communicating, staying healthy, nourishment, safety, loneliness/social network, and psychosocial problems.			2. First Step. Social network, patient has: Sufficient and strong social network; Large but weak social network; Small but strong social network; Small and weak or no social network; Unknown<br>3. Second Step. Do you live alone? (No/Yes)<br>4. Second Step. Is there anyone who would be able to help you in case of illness or emergency? (No/Yes)<br>5. Second Step. Do you have contact with people in your neighbourhood? With few people; little contact; With few people, but sufficient contact; With many people, little contact; With enough people, sufficient contact.<br>6. Second Step. Do you feel lonely? Never; Sometimes; Often.
11	Garre-Olmo, et al. (2013)	The Social Frailty Phenotype, SPF	The SPF includes 6 deficits that are based on deficit accumulation in the absence of disability. Operational definitions of social frailty consisted of indicators that are rated as present or absent. Participants who meet 2 or more deficits are considered as SF.	6	6	1. Living alone.<br>2. Absence of a person to help with ADL: Do you have family and/or friends you could ask for help if you needed assistance?<br>3. Infrequent family contact: How often do you meet or talk to your closest relatives (every day, every 2 or 3 days, weekly, monthly, once a year)?<br>4. Infrequent friend/neighbour contact: How often do you meet or talk to your friends and/or neighbors (every day, every 2 or 3 days, weekly, monthly, once a year)?<br>5. Absence of a confident: Is there anyone special (a couple, friend, family member, and/or neighbor) that you can trust and talk to about personal matters and your feelings?<br>6. Lack of support for daily living: In the past 3 months, have you failed to receive help from others with shopping, food preparation, housecleaning, ironing or other personal activities even though you needed help?

12	Tavassoli, et al. (2014)	The Gérontopôle Frailty Screening Tool, GFST	The GFST is composed of 6 items corresponding to domains of living alone, weight loss, fatigue, mobility, memory, and slowness.	6	1	1. Does your patient live along?
13	Makizako, et al. (2015)	Questionnaire to define Social Frailty Status, QSFS	QSFS is composed of 7 questions regarding daily social activity, social role, and social relationships for determination of social frailty status. After analysis, 5 questions are kept. Participants who meet 0 criterion is considered as non-SF, meeting one criterion is pre-SF, and meeting two or more is considered as SF.	5	5	1. Do you go out less frequently compared with last year? (yes)<br>2. Do you sometimes visit your friends? (no)<br>3. Do you feel you are helpful to friends or family? (no)<br>4. Do you live alone? (yes)<br>5. Do you talk with someone every day? (no)
14	Satake, et al. (2016)	The Kihon Checklist, KCL	The KCL is composed of 25 questions regarding instrumental, social activities of daily living, physical functions, nutritional status, oral function, cognitive function, and depressive mood.<br>The items are representing with yes/no answer. In the KCL, with a higher score in each domain of the checklist indicates higher risk of requiring support or care in that domain.	25	4	1. Do you sometimes visit your friends?<br>2. Do you turn to your family or friends for advice?<br>3. Do you go out at least once a week?<br>4. Do you go out less frequently compared to last year?
15	Vernerey, et al., (2016)	The Frailty Groupe Iso Ressource Evaluation, FRAGIRE	The FRAGIRE is composed of 19 items describing 9 dimensions (global health status, psychological, cognitive, environmental, socio-cultural, sexual, burden of help, nutritional, and mobility dimension).	19	4	1. SOCIO-CULTURAL: Do you use Internet?<br>2. SOCIO-CULTURAL: Do you participate in sport activities (sport, art, etc.)?<br>3. ENVIRONMENTAL: Have you felt lonely or abandonment?<br>4. ENVIRONMENTAL: Your financial situation seemed sufficient to meet your needs?
16	Morris, et al. (2016)	The interRAI Home Care Frailty Scale, IRAHCFS	The IRAHCFS is composed of 29 items. The items fall across 6 categories of function, movement, cognition and communication, social life, nutrition, and clinical symptoms.	29	3	1. Social: Decline in social activities.<br>2. Social: Reduced social activities.<br>3. Social: Withdrawal From Activities of Interest.
17	Teo, et al. (2017)	The Social Frailty Index, SFI	The SFI is assessed through a 7-questions index including living alone, no education, absence of a confident, infrequent contact, infrequent social activities, financial difficulty and socioeconomic depravation. Scores are assigned to each SF indicator (1 = present, 0 =	7	7	1. Living alone: Who do you live with?<br>2. No education: What is your education level?<br>3. Absence of a confidant: Do you have someone to confide in?<br>4. Infrequent contact:(a) none or once a year visits from family,

			absent), and the summed scores are used to categorize individuals as having high score (2–7), low score (1), and nil score (0 point) on the SF index.			friends or loved ones; (b) none or once a year calls from family, friends or loved ones or (c) none to a very little extent of help when they require it.<br>5. Infrequent social activities [(1) Attendance at any religious service; (2) visits to cinemas, restaurants or sports events; (3) day or excursion trips; (4) if they play cards, games, bingo, mahjong; (5) if they attend senior citizen club activities or (6) if they attend social group activities.)]<br>6. Financial difficulty: Are you limited by your financial resources to pay for needed medical service?<br>7. Social economic deprivation (1-2 room flats & others).
18	Ma, et al. (2018)	The HALFT scale	"HALFT" is an acronym for the five components: Help, pArticipation, Loneliness, Financial and Talk. The HALFT scale is composed of 5 items: inability to help others, limited social participation, loneliness, financial difficultly, and not having anyone to talk to. The score ranges from 0–5 points: a score of 0 is considered as non-SF; 1–2 is considered as pre-SF; and a score of ≥3 indicates SF.	5	5	1. I was able to help friends or family within the past 12 months.<br>2. I had engaged in any social or leisure time activities in the previous 12 months.<br>3. I have felt lonely in the past week<br>4. Income was enough for living over the past 12 months.<br>5. I have someone who I can talk with every day.
19	Yamada, et al. (2018)	The social frailty screening questionnaire, SFSQ	The SFSQ is developed by a group of medical doctors, physical therapists, occupational therapists, and public health nurses. The questionnaire based on Bunt's social frailty concept, which includes general resources, social resources, social behavior, and the fulfillment of basic social needs. The answers which are "very unsatisfied with economic condition", "living alone", "participating in none of these activities", and "a neighbor to say hello to, and do not communicate with neighbors" are considered as poor resources.	4	4	1. general resources (financial difficulties): Are you satisfied with your economic condition?<br>2. social resources (living alone): Do you live alone?<br>3. social behavior (lack of social activity) : Which social activities do you participate in (multiple answers allowed)?<br>4. fulfillment of basic social needs (influential contact with neighbors): How do you get along with your neighbors?
20	Yoo, et al. (2019)	Social frailty, SF-Korea	The social frailty (Korea) includes five components which are validated to predict disability from previous studies. Answer of "yes" to the questions 4 and "no" to the questions	5	5	1. Do you attend neighborhood meetings, social gatherings such as senior citizens' welfare centers, clubs, classes, etc.

			1,2,3,5 are considered as SF indicator. A total score of 0 is classified as non-SF, that of 1or 2 is pre-SF, and that of 3 or more is SF.			2. Do you sometimes meet or talk to your friend(s)?<br>3. Is there someone available for you and who shows you love and affection?<br>4. Do you live alone?<br>5. Do you have any family, relatives or neighbors who meet or talk on the phone every day?
21	Pek, et al. (2020)	Social Frailty	The items identified from published Asian studies (Makizako, et al., 2015; Tsutsumimoto, et al., 2017; Tanaka, et al., 2017; Teo, et al., 2017)were used as social frailty questionnaire items. The combined 9-item social frailty questionnaire was administered, with equal weightage of one point assigned to each item.	9	9	1. Do you live alone?<br>2. Do you go out less frequently compared with last year?<br>3. Do you sometimes visit your friends?<br>4. Do you feel you are helpful to friends or family?<br>5. Do you talk with someone every day?<br>6. Do you turn to family or friends for advice?<br>7. Do you eat with someone at least one time in a day?<br>8. Do you have someone to confide in?<br>9. Are you limited by your financial resources to pay for needed medical service?
22	Chen, et al. (2021)	Social Frailty	Six questions representing family disharmony, living alone, talking to someone, visiting friends, feeling lonely, and financial difficulty were extracted from past studies. participants showing none or one of these components were considered non-socially frail; those showing 2 or 3 components were considered pre-socially frail; and those showing 4 or more components were considered socially frail.	6	6	1. Is your family in harmony?<br>2. Are you living alone now?<br>3. When faced with troubles, do you have anyone to talk to?<br>4. In the past week, have you visited your friends, or have your friends come to visit you?<br>5. Do you feel lonely?<br>6. Financial difficulty was determined by membership in the lowest tertile of the wealth index.
23	Ragusa, et al. (2022)	Social frailty	Four components (financial difficulty, household status, social activity, and contacts with other people) which defined social frailty in the dataset of ELSA study were included. For each component, two values (0 less severe and 1 more severe) were attributed, resulting in a final score from zero to four. The total score was then divided into social frailty (≥2 points), social pre-frailty (1 point), and robustness (0 points)	4	4	1. Financial difficulty was defined using the threshold for poverty of the UK population, considering the total family level income below £20,346<br>2. household status (living alone vs. not living alone)<br>3. social activity (non-participation in social activities vs. participation in social activities)<br>4. contacts with other people, defined as weekly contact with friends in person/phone/email

24	Lee, et al. (2024)	Social frailty	Social frailty was operationalized and assessed at baseline using the 5 domains guided by Bessa et al: social support, social activity, social network, loneliness, and living alone. The total ranged from 0 to 5, and we categorized 2 or more points as social frailty, 1 point as social prefrailty, and 0 point as social nonfrailty.	5	5	1. Social support: Except for your spouse and those who live with you, do you have any friends or relatives who can help you if you are unable to carry out your daily life?<br>2. Do you participate in any activities? (e.g., religious group, social club such as alumni group or senior center, leisure/cultural/sport group, volunteering, political/civic/interest group)<br>3. Social network: Do you have any close friends or relatives?<br>4. Did you feel lonely during the past week?<br>5. Do you live alone?
Frailty Index						
25	Jones, et al. (2005)	The Frailty Index Comprehensive Geriatric Assessment, FI-CGA	The FI-CGA is the sum of the impairment index and co-morbidity index scores. As the maximum impairment index score is 10 and that of the co-morbidity index is 4, the largest number of potential accumulated deficits is 14 as FI-CGA=1. Each FI-CGA value is thus normalized to the range of 0 to 1.	14	1	1. Social: Institutionalized; Uses formal home supports; Living alone (Deficit)
26	de Vries, et al., (2013)	Evaluative Frailty Index for Physical Activity, FI-EFIPA	This FI-EFIPA is comprised of 50 deficits across general health status, physical, psychological, and social domains.	50	7	1. Do you feel lonely?<br>2. When you need help, are there people who are willing and able to help you?<br>3. Are there activities that someone else has taken over for you recently?<br>4. Are there enough organized activities for you nearby?<br>5. Do you have problems getting out for organized activities (e.g., problems with transportation to get to them)?<br>6. Do you have any housing problems?<br>7. Do you have enough help from professionals?
27	Myers, et al. (2014)	The Rockwood index of accumulation of deficits (1), FI-1	The Rockwood index of accumulation of deficits is using by Myers, et al. (2014). This index is comprised of 40 deficits across perceived health, comorbidity, functional limitations, weight loss, physical activity and psychological factors. Dichotomous items are coded as 0 if the deficit is absent and	40	4	1. Work limitations<br>2. Social activities limitations<br>3. Leisure time physical activity<br>4. Loneliness.

			1 if it is present; while ordinal or continuous variables are graded into a score between 0 and 1 (0 for no impairment, 0.5 for minor impairment and 1 for major impairment). Scores are summed and divided by the total number of variables to give a frailty index between 0 and 1, with 1 representing the greatest frailty.			
28	McKenzie, et al. (2015).	The Frailty Index(2), FI-2	This FI is comprised of 42 deficits across physiological, psychological, cognitive, social, and service use domains.	42	3	1. Changes in social activities<br>2. Social isolation<br>3. Loneliness
29	Ma, et al. (2016)	The Frailty Index(3), FI-3	This FI is comprised of 68 parameters across aspects of demographic characteristics, physical health, physical function, living behavior, social function, mental health, and cognitive function.	68	6	1. Smoking and drinking<br>2. Participation in social activities<br>3. Physical exercise<br>4. Work<br>5. Housework<br>6. Sleep quality
30	Yamanashi, et al. (2015)	The Frailty Index(Japan), FI-J	Index for Japanese elderly (FI-J) is comprised of 15 items relating with “frequency of going out”, “fall history”, and “malnutrition”.	15	5	1. Lower daily physical activity<br>2. Less outdoor activity<br>3. Fewer hobbies or interests<br>4. Less contact with neighbours<br>5. Less friendships other than neighbours
31	Kwan, et al. (2015)	The Comprehensive Model of Frailty, CMF	Kwan, et al. (2015) create a 32-item FI (FI- 32) that represented deficits in a range of systems including chronic diseases, fatigue, mobility restrictions, sensory loss, difficulties in activities of daily living, memory problems, and negative emotions. They then create the CMF adding 12 items that assess the deficits in the psychological, social/family, environmental and economic domains to FI- 32.	44	5	1. Social/family factors: Living alone or with their family members.<br>2. Social/family factors: Frequency of attending social activities.<br>3. Social/family factors: Having a spouse or a child to confide with when they need emotional support.<br>4. Environmental factors: Barriers to social activities.<br>5. Economic factors: Perceived self-rated economic status.
32	Young, et al. (2016)	The Frailty Index(4), FI-4	This FI is comprised of 39 parameters across the domains of comorbid conditions, physical measures, biochemical measures, mental health, self-reported general health, disability, social functioning, polypharmacy, and pain.	39	2	1. Social network (During the past 4 weeks, to what extent has your physical health or emotional problems interfered with your normal social activities with family, friends, neighbours, or groups?)<br>2. During the past 4 weeks, how much of the time has your physical health or emotional problems interfered with your social activities - like visiting friends, relatives, etc.?)

33	Bäckman, et al. (2017)	The Frailty Index(5), FI-5	This FI is comprised of 38 deficits relating with a range of systems of age and health.	38	2	1. Do you feel lonely?<br>2. Daily contact with other people through meetings, phone, contacts, emails, etc.
34	Dent, et al. (2017)	The Frailty Index(6), FI-6	This FI is comprised of 35 deficits across self-reported medical conditions, function, disease history, health and psychosocial factors.	35	6	1. Problems with transport when you want to go out<br>2. Activities limited because of any impairment of health problem<br>3. Marital status<br>4. Family´s money situation<br>5. Current family structure (lives alone)<br>6. There are people in my life that really care about me
35	Lee, et al. (2020)	Social deficits	Social deficits were defined as deficiencies in socioeconomic status, living situation, social network size, contact with social networks, social support, and social activities. The number of social deficits was categorized as none (0), low (1-2), and high (3 or more), based on the summated score. A social deficit score was calculated summing the 9 variables, with the social deficit level categorized as none (0, lower quartile), low (1-2), and high (3-9, upper quartile). The median values of 2 and 3 on the social deficit score corresponded to prefrail and frail, respectively, on the frailty status.	9	9	1. Socioeconomic status included education and household income.<br>2. The living situation included marital status and family structure.<br>3. The social network included number (none) of close relatives, friends, or neighbors and the frequency of contact (rarely).<br>4. For social support, participants were asked about emotional (listening to concerns or worries), instrumental (help with housework, preparing meals, and doing laundry), and care (caregiving during illness) support received from or provided to family members, relatives, friends, or neighbors.<br>5. Social participation covered engagement in 8 types of activities.
36	Shah, et al. (2023)	Social Frailty Index	The Social Frailty Index includes age, gender, and eight social characteristics and accurately risk-stratifies older adults.	10	10	1. How old are you?<br>2. What is your gender?<br>3. Are you currently working for pay?<br>4. Do you have any living children?<br>5. Thinking about all of your living children. How often do you meet up(include both arranged and chance meetings)<br>6. Please tell us how often you do each activity.<br>7. How much of the time do you feel isolated from others?

						8. How you feel about your local area?<br>9. How would you rate the amount of control you have over your financial situation?<br>10. How often have any of the following things happened to you? (you are treated with less courtesy or respect than other people.)
37	Irshad, et al. (2024)	Social Frailty Indicators	For the assessment of social frailty the study proposed a multidimensional Social Frailty Index (SFI) score ranging between 0 and 100 using 17 indicators. Data from the Longitudinal Ageing Study in India (LASI)-wave 1 was used.	17	17	1. Close relationship with family/relative/others<br>2. Have friends<br>3. Frequency of meeting friends<br>4. Frequency of connecting friends over phone or mail/e-mail<br>5. Share personal matters with family/friends/others<br>6. Membership and participation in social organisation<br>7. Decision making participation on marriage of son/daughter)<br>8. Decision making participation on buying and selling of property<br>9. Decision making participation on gifts to children, grandchildren and other relatives<br>10. Decision making participation on education of family member<br>11. Decision making participation on arrangement of social/religious events<br>12. Eat out of the house (restaurant/hotel)<br>13. Go to park/beach for relaxing/entertainment<br>14. Play cards or indoor games<br>15. Play outdoor games/sports/exercise/jog/yoga<br>16. Attend religious functions<br>17. Attend political/community/organisation group meetings
Social Vulnerability Index						
38	Andrew, et al. (2008)	The Social Vulnerability Index, SVI-CA-2008	This social vulnerability index is composed of 40 items covering domains such as social support, social engagement, sense of mastery/control over one's life circumstances, health status, and socioeconomic status.	40	40	1. Read English or French<br>2. Write English or French<br>3. Marital status<br>4. Lives alone<br>5. Someone to count on for help or support<br>6. Feel need more help or support

						7. Someone to count on for transportation<br>8. Feel need more help with transportation<br>9. Someone to count on for help around the house<br>10. Feel need more help around the house<br>11. Someone to count on to listen<br>12. Feel need more people to talk with<br>13. Number of people spend time with regularly<br>14. Feel need to spend more time with friends/family<br>15. Someone to turn to for advice<br>16. Feel need more advice about important matters<br>17. Telephone use<br>18. Get to places out of walking distance<br>19. How often visit friend or relatives<br>20. How often work in garden<br>21. How often golf of play other sports<br>22. How often go for a walk<br>23. How often go to clubs, church, community centre<br>24. How often play cards or other games<br>25. Feel empowered, in control of life situation<br>26. Maintaining close relationships is difficult and frustrating<br>27. Experience of warm and trusting relationships<br>28. People would describe me as a giving person<br>29. Family relationships<br>30. Friendships<br>31. Housing<br>32. Finances<br>33. Neighbourhood<br>34. Activities<br>35. Religion<br>36. Transportation<br>37. Life generally<br>38. Does income currently satisfy needs<br>39. Home ownership<br>40. Education
39	Andrew &	The Social	This social vulnerability index is composed of 28 items covering	28	28	1. Frequency of group engagement

	Keefe (2014)	Vulnerability Index, SVI-CA-2014	individual-level social variables (engagement, contextual SES, social support, living situation, self-esteem, sense of control, and relations with others) and five neighbourhood-level variables. Each variable is coded in terms of potential social "deficits" such that respondents were assigned a score of 0 if the deficit is absent and 1 if it is endorsed, with intermediate values apply in the case of ordered response categories. Vulnerability on each item is mapped to the 0-1 interval and the scores are summed and divided by the total number of deficits to create a summary social vulnerability index.			2. No group engagement<br>3. Attending religious services<br>4. Physical leisure activities<br>5. EA: income<br>6. EA: education<br>7. EA: inequality<br>8. EA: unemployment<br>9. Support: advice<br>10. Support: help in a crisis<br>11. Support: someone to confide in<br>12. Support: someone to make you feel loved<br>13. Frequency of contact with relatives<br>14. Lives alone<br>15. Marital status<br>16. Worth equal to others<br>17. Positive attitude towards self<br>18. Too much expected of you by others<br>19. Want to move but cannot<br>20. Not enough money to buy the things you need<br>21. Little control over things that happen<br>22. How often have people let you down<br>23. Noisy/polluted neighbourhood<br>24. Frequency of contact with neighbours<br>25. EA: caregiving for seniors<br>26. Frequency of contact with friends<br>27. Education<br>28. Ability to speak English or French
40	Armstrong, et al. (2015)	Social Vulnerability Index, SVI-USA	This social vulnerability index is composed of 19 items covering domains such as marital status, living situation, social supports and social engagement. Each variable is dichotomized with values of '1' representing the presence of a social deficit and '0' marking the absence.	19	19	1. When lonely, there is no one to talk to<br>2. I rarely meet/talk with family or friends<br>3. Cannot find somebody help w/ daily chore<br>4. Do not know somebody that can turn to with personal problem<br>5. Do not trust at least one person's advice<br>6. Could not find someone to care for house<br>7. Do not have somebody to talk to about important decisions<br>8. People do not talk to you about important decisions<br>9. Don't have people to help with things like shopping, etc.

						10. Living alone<br>11. See few many relatives once a month<br>12. Feel close to few people or relatives<br>13. No close friends<br>14. Do not participate in any groups<br>15. Hearing cause difficulty when visiting friends<br>16. Unable to see to recognize friend across a street<br>17. Do not do regular volunteer work<br>18. Present marital status<br>19. Low yearly household income
Other						
41	van Oostrom, et al. (2017)	Rasch-type loneliness scale	Rasch-type loneliness scale is developed by De Jong-Gierveld & Kamphuis (1985). The scale includes aspects of both emotional and social loneliness, and it consists of six negatively and five positively formulated items. The Rasch-type loneliness scale is used to defined social frailty in van Oostrom, et al.'s study (2017).	11	11	1. Often, I feel rejected.<br>2. I miss having a really close friend.<br>3. I feel my circle of friends and acquaintances is too limited.<br>4. I miss having people around.<br>5. I miss the pleasure of the company of others.<br>6. I experience a general sense of emptiness.<br>7. There are plenty of people that I can lean on in case of trouble.<br>8. There is always someone that I can talk to about my day-to-day problems.<br>9. I can call on my friends whenever I need them.<br>10. There are enough people that I feel close to.<br>11. There are many people that I can count on completely.
42	van Oostrom, et al. (2017)	Social Support List-Interactions, SSL12-I	The SSL 12-I (Kempen, et al., 1995) consists of 12 items, divided into three subscales: everyday support, social support in problem situations, and esteem support.<br>The SSL 12-I is used to defined social frailty in van Oostrom, et al.'s study (2017).	12	12	1. invite you to a party or to dinner?<br>2. drop in for a (pleasant) visit?<br>3. show you that they are fond of you?<br>4. are interested in you?<br>5. comforting you<br>6. provide you with help in special circumstances, such as: illness, or moving home?<br>7. reassure you?<br>8. give you good advice?<br>9. pay you a compliment?<br>10. confide in you?<br>11. ask you for help or advice?<br>12. emphasize your strong points?

43	Liu, et al. (2024)	Social frailty	Social frailty was assessed by Loneliness and social isolation index. Loneliness was measured using a widely used single question from the Centre for Epidemiological Studies Depression scale (CESD). A total score of social isolation index ranges from 0 to 6, with a higher score indicating greater social isolation The final score of social frailty is the sum of 1 point for loneliness and 6 points for social isolation, and ranges from 0 to 7, with higher scores indicating greater social frailty.	7	7	1. how often you felt lonely during the last week? Participants were assigned one point if<br>2. they did not get married,<br>3. not have weekly contact with children in person,<br>4. not have weekly contact with children by phone or e-mail,<br>5. not participate in any social activity (e.g. interact with friends; play chess or cards; go to a sport, social or other clubs),<br>6. reside in rural areas,<br>7. live alone

1. J. Barber, J. B. Wallis, E. McKeating, A postal screening questionnaire in preventive geriatric care. The Journal of the Royal College of General Practitioners 30, 49-51 (1980).
2. 2.1 F. Cacciatore et al., Frailty predicts long-term mortality in elderly subjects with chronic heart failure. European journal of clinical investigation 35, 723-730 (2005).
   2.2 M. S. Lachs et al., A simple procedure for general screening for functional disability in elderly patients. Annals of internal medicine 112, 699-706 (1990).
3. R. Hébert, G. Bravo, N. Korner-Bitensky, L. Voyer, Refusal and information bias associated with postal questionnaires and face-to-face interviews in very elderly subjects. Journal of clinical epidemiology 49, 373-381 (1996).
4. D. Saliba et al., The Vulnerable Elders Survey: a tool for identifying vulnerable older people in the community. Journal of the American Geriatrics Society 49, 1691-1699 (2001).
5. H. Schuurmans, N. Steverink, S. Lindenberg, N. Frieswijk, J. P. Slaets, Old or frail: what tells us more? The Journals of Gerontology Series A: Biological Sciences and Medical Sciences 59, M962-M965 (2004).
6. D. B. Rolfson, S. R. Majumdar, R. T. Tsuyuki, A. Tahir, K. Rockwood, Validity and reliability of the Edmonton Frail Scale. Age and ageing 35, 526-529 (2006).
7. M. Raîche, R. Hébert, M.-F. Dubois, PRISMA-7: a case-finding tool to identify older adults with moderate to severe disabilities. Archives of gerontology and geriatrics 47, 9-18 (2008).
8. R. J. Gobbens, M. A. van Assen, K. G. Luijkx, M. T. Wijnen-Sponselee, J. M. Schols, The Tilburg frailty indicator: psychometric properties. Journal of the American Medical Directors Association 11, 344-355 (2010).
9. N. De Witte et al., The comprehensive frailty assessment instrument: development, validity and reliability. Geriatric Nursing 34, 274-281 (2013).
10. J. A. van Kempen et al., Development of an instrument for the identification of frail older people as a target population for integrated care. British Journal of General Practice 63, e225-e231 (2013).
11. J. Garre-Olmo, L. Calvó-Perxas, S. López-Pousa, M. de Gracia Blanco, J. Vilalta-Franch, Prevalence of frailty phenotypes and risk of mortality in a community-dwelling elderly cohort. Age and ageing 42, 46-51 (2013).
12. N. Tavassoli et al., Description of 1,108 older patients referred by their physician to the “Geriatric Frailty Clinic (GFC) for Assessment of Frailty and Prevention of Disability” at the gerontopole. The

Journal of nutrition, health and aging 18, 457-464 (2014).
13. H. Makizako et al., Social frailty in community-dwelling older adults as a risk factor for disability. Journal of the American Medical Directors Association 16, 1003. e1007-1003. e1011 (2015).
14. S. Satake et al., Validity of the K ihon Checklist for assessing frailty status. Geriatrics & gerontology international 16, 709-715 (2016).
15. D. Vernerey et al., Development and validation of the FRAGIRE tool for assessment an older person's risk for frailty. BMC geriatrics 16, 1-28 (2016).
16. J. N. Morris, E. P. Howard, K. R. Steel, Development of the interRAI home care frailty scale. BMC geriatrics 16, 1-9 (2016).
17. N. Teo, Q. Gao, M. S. Z. Nyunt, S. L. Wee, T.-P. Ng, Social frailty and functional disability: findings from the Singapore longitudinal ageing studies. Journal of the American Medical Directors Association 18, 637. e613-637. e619 (2017).
18. L. Ma, F. Sun, Z. Tang, Social frailty is associated with physical functioning, cognition, and depression, and predicts mortality. The Journal of nutrition, health and aging 22, 989-995 (2018).
19. M. Yamada, H. Arai, Social frailty predicts incident disability and mortality among community-dwelling Japanese older adults. Journal of the American Medical Directors Association 19, 1099-1103 (2018).
20. M. Yoo et al., Moderate hearing loss is related with social frailty in a community-dwelling older adults: The Korean Frailty and Aging Cohort Study (KFACS). Archives of gerontology and geriatrics 83, 126-130 (2019).
21. K. Pek et al., Social frailty is independently associated with mood, nutrition, physical performance, and physical activity: Insights from a theory-guided approach. International journal of environmental research and public health 17, 4239 (2020).
22. Z. Chen et al., Social frailty and longitudinal risk of depressive symptoms in a Chinese population: the Rugao longevity and aging study. Psychogeriatrics 21, 483-490 (2021).
23. F. S. Ragusa et al., Social frailty increases the risk of all-cause mortality: A longitudinal analysis of the English Longitudinal Study of Ageing. Experimental gerontology 167, 111901 (2022).
24. J. J. Lee, M. K. Park, N. Kim, L. Kim, G. S. Kim, Longitudinal relationship between baseline social frailty and cognitive impairment in older adults: 14-Year follow-up results from the Korean Longitudinal Study of Ageing. Journal of the American Medical Directors Association 25, 105124 (2024).
25. D. Jones, X. Song, A. Mitnitski, K. Rockwood, Evaluation of a frailty index based on a comprehensive geriatric assessment in a population based study of elderly Canadians. Aging clinical and experimental research 17, 465-471 (2005).
26. N. M. De Vries, J. B. Staal, M. G. Olde Rikkert, M. W. Nijhuis-van der Sanden, Evaluative frailty index for physical activity (EFIP): a reliable and valid instrument to measure changes in level of frailty. Physical therapy 93, 551-561 (2013).
27. V. Myers, Y. Drory, U. Goldbourt, Y. Gerber, Multilevel socioeconomic status and incidence of frailty post myocardial infarction. International journal of cardiology 170, 338-343 (2014).
28. K. McKenzie, H. Ouellette-Kuntz, L. Martin, Using an accumulation of deficits approach to measure frailty in a population of home care users with intellectual and developmental disabilities: an analytical descriptive study. BMC geriatrics 15, 1-13 (2015).
29. L. Ma et al., Use of the frailty index in evaluating the prognosis of older people in Beijing: a cohort study with an 8-year follow-up. Archives of gerontology and geriatrics 64, 172-177 (2016).

30. H. Yamanashi et al., The association between living alone and frailty in a rural Japanese population: the Nagasaki Islands study. Journal of primary health care 7, 269-273 (2015).
31. J. S. K. Kwan, B. H. P. Lau, K. S. L. Cheung, Toward a comprehensive model of frailty: an emerging concept from the Hong Kong centenarian study. Journal of the American Medical Directors Association 16, 536. e531-536. e537 (2015).
32. A. C. Young, K. Glaser, T. D. Spector, C. J. Steves, The identification of hereditary and environmental determinants of frailty in a cohort of UK twins. Twin Research and Human Genetics 19, 600-609 (2016).
33. K. Bäckman et al., Changes in the lethality of frailty over 30 years: evidence from two cohorts of 70-year-olds in Gothenburg Sweden. Journals of Gerontology Series A: Biomedical Sciences and Medical Sciences 72, 945-950 (2017).
34. E. Dent, E. Dal Grande, K. Price, A. W. Taylor, Frailty and usage of health care systems: Results from the South Australian Monitoring and Surveillance System (SAMSS). Maturitas 104, 36-43 (2017).
35. Y. Lee, D. Chon, J. Kim, S. Ki, J. Yun, The predictive value of social frailty on adverse outcomes in older adults living in the community. Journal of the American Medical Directors Association 21, 1464-1469. e1462 (2020).
36. S. J. Shah et al., Social Frailty Index: Development and validation of an index of social attributes predictive of mortality in older adults. Proceedings of the National Academy of Sciences 120, e2209414120 (2023).
37. C. Irshad, D. Govil, H. Sahoo, Social frailty among older adults in India: Findings from the Longitudinal Ageing Study in India (LASI)–Wave 1. Experimental Aging Research 50, 331-347 (2024).
38. M. K. Andrew, A. B. Mitnitski, K. Rockwood, Social vulnerability, frailty and mortality in elderly people. PloS one 3, e2232 (2008).
39. M. K. Andrew, J. M. Keefe, Social vulnerability from a social ecology perspective: a cohort study of older adults from the National Population Health Survey of Canada. BMC geriatrics 14, 1-14 (2014).
40. J. J. Armstrong et al., Social vulnerability and survival across levels of frailty in the Honolulu-Asia Aging Study. Age and ageing 44, 709-712 (2015).
41. S. H. van Oostrom et al., A four-domain approach of frailty explored in the Doetinchem Cohort Study. BMC geriatrics 17, 1-11 (2017).
42. S. H. van Oostrom et al., A four-domain approach of frailty explored in the Doetinchem Cohort Study. BMC geriatrics 17, 1-11 (2017).
43. Q. Liu et al., Joint trajectories of physical frailty and social frailty and associations with adverse outcomes: A prospective cohort study. Archives of Gerontology and Geriatrics 122, 105406 (2024).

Table S3: Classification of social components

	Social components	Instruments (N=43)	Items (N=298)
**1**	**Social support**	**23**	**63**
**2**	**Social participation**	**28**	**53**
**3**	**Social network**	**23**	**40**
**4**	**Loneliness**	**16**	**33**
5	Living along	20	20
6	Socio-demographic	19	44
7	Social role	4	8
8	Socially oriented Activities of Daily Living	2	3
9	Communication to engage in wider community	2	3
10	Work	2	3
11	Social context	1	1
12	Feel about life	2	10
13	Lifestyle	1	3
14	Self-esteem	1	2
15	Sense of control	2	8
16	Ryff scale	1	4

Table S4: Item-analysis

No	Items	1.missing data	2.mean (non-sig)	3.standard deviation (>0.5)	4.skewness (< ±3)	5.kurtosis (< ±10)	6.comparisons of extreme groups (sig)	7.correlation coefficient of items (>.32)	8.factor loading (>.32)	Cumul-ation
1	Is there someone caring about you?	0	.000	.69	1.42	1.46	-7.14	.45	.47	1
2	Do you have someone to confide in (e.g. a spouse, family members, friends, and/or neighbors) ?	0	.307	.96	1.01	-.17	-7.90	.46	.52	0
3	Is there someone comforting you?	0	.001	.85	1.16	.42	-9.73	.55	.61	1
4	Is there someone paying you a compliment?	0	.045	.95	0.53	-.91	-12.09	.59	.64	1
5	Is there someone emphasizing your strong points?	0	.011	.98	0.48	-.99	-12.39	.57	.60	1
6	Do you have someone you could ask for help if you needed assistance? (e.g. shopping, housework…)	0	.792	1.02	0.90	-.46	-8.64	.50	.55	0
7	Do you have someone you could ask for help if you were in case of illness or emergency (e.g. sudden fall)?	0	.001	.88	1.31	.78	-8.00	.47	.51	1
8	Do you have someone you could ask for help if you were in a crisis?	0	.018	.91	1.18	.33	-8.65	.53	.56	1
9	Do you have someone you could ask for help if you need help with transportation?	0	.027	.92	1.18	.38	-7.79	.46	.50	1
10	Do you have enough help from professionals for advice (e.g. medical,	0	.073	.99	0.55	-1.01	-8.27	.46	.49	0

	law, finance, and/ or social welfare)?									
11	Do you turn to your family, relatives or friends when you need some advice?	0	.724	.95	0.82	-.41	-9.43	.57	.62	0
12	How often do you participate in social activities? (e.g. family/friends gathering, community activities…)	0	.000	.78	1.65	2.17	-8.33	.54	.57	1
13	How often do you participate in community activities? (e.g. congregate meal, traveling, classes…)	0	.038	.88	1.05	.12	-7.89	.47	.51	1
14	How often do you paly card or other puzzle games? (e.g. mah-jong, Chinese chess, board game…)	0	.000	1.19	-0.70	-1.13	-6.83	.33	.34	1
15	How often do you participate in group activities? (e.g. clubs, learning activities…)	0	.691	.98	1.00	-.16	-8.77	.49	.52	0
16	How often do you participate in religious activities? (e.g. religious event, worship…)	0	.000	1.14	-0.50	-1.19	-7.26	.36	.41	0
17	How often do you participate in physical activities? (e.g. exercise, take a walk, outdoor activities…)	0	.797	1.01	0.99	-.25	-7.96	.45	.48	0
18	How often do you contact with family through line/FB, email, etc.?	0	.000	.79	1.99	3.24	-5.54	.36	.38	1
19	How often do you meet your family?	0	.000	.73	2.05	3.60	-6.00	.33	.34	1

20	How often do you contact with friends through line/FB, email, etc.?	0	.626	.96	0.87	-.28	-12.10	.60	.62	0
21	How often do you meet your friends ?	0	.787	.96	0.97	-.13	-7.93	.50	.53	0
22	How often do you contact with neighbors through line/FB, email, etc.?	0	.850	1.02	0.98	-.33	-5.43	.36	.39	0
23	How often do you meet your neighbors ?	0	.000	.85	1.49	1.32	-5.41	.39	.41	1
24	Do you feel lonely?	0	.000	.69	2.08	4.43	-4.10	.26	.21	3
25	Do you feel rejected?	0	.000	.55	2.291	6.45	-3.13	.21	.16	3
26	Do you miss having people around you?	0	.000	1.21	-.017	-1.58	0.214	-.11	-.15	4
27	Do you feel sense of emptiness?	0	.000	.76	2.138	4.17	-2.76	.20	.13	3
28	Do you feel abandonment?	0	.000	.62	3.400	11.42	-2.73	.21	.14	5
29	Do you feel sense of social isolation?	0	.000	.72	2.893	7.61	-2.05	.09	.03	3

Note: The criteria used were: 1) no missing data; 2) t-tests result shall show that the mean of each item and that of total scale are not significant; 3) standard deviation of each items shall be higher than 0.5 ($m_{2abs} = \frac{X_4 - X_1}{6}$); 4) The absolute value of skewness of each item shall be less than 3; 5)and the absolute value of kurtosis shall be less than 10; 6) the result of comparisons of extreme groups shall be significant; and 7) The correlation coefficient of items shall be higher than 0.32; 8) factor loading shall be higher than 0.32. Items that meet over three of these criteria were excluded.

H. J. Chiou, *Quantitative Research Method: Principles and Techniques of Testing and Scale Development* (Yehyeh book, Taipei, 2018).

Table S5: Analysis of confirmatory factor analysis and reliability (N =219)

item	standardized factor loading	SMC	component reliability	AVE
1. Is there someone caring about you?	0.803	0.645	0.791	0.559
2. Do you have someone to confide in (e.g. a spouse, family members, friends, and/or neighbors) ?	0.659	0.434		
3. Is there someone comforting you?	0.774	0.599		
7. Do you have someone you could ask for help if you were in case of illness or emergency (e.g. sudden fall)?	0.863	0.745	0.813	0.599
8. Do you have someone you could ask for help if you were in a crisis?	0.851	0.725		
9. Do you have someone you could ask for help if you need help with transportation?	0.572	0.327		
12. How often do you participate in social activities? (e.g. family/friends gathering, community activities…)	0.755	0.57	0.799	0.570
13. How often do you participate in community activities? (e.g. congregate meal, traveling, classes…)	0.766	0.587		
15. How often do you participate in group activities? (e.g. clubs, learning activities…)	0.743	0.553		
20. How often do you contact with friends through line/FB, email, etc.?	0.511	0.261	0.784	0.492
21. How often do you meet your friends?	0.484	0.234		
22. How often do you contact with neighbors through line/FB, email, etc.?	0.863	0.745		
23. How often do you meet your neighbors?	0.854	0.729		
24.Do you feel lonely?	0.880	0.775	0.779	0.550
25.Do you feel rejected?	0.522	0.272		
27.Do you feel sense of emptiness?	0.777	0.604		

*Note1:*The criterion for component reliability is greater than 0.60, the average variance extracted (AVE) is greater than 0.50.

M. L. Wu, *Structural Equation Modeling with AMOS* (Wunan, Taipei, 2009).

Table S6: Analysis of criterion-related validity (N=446)

NO	SCALE	1	2	3	4	5	6
1	Total score of social frailty (16items)	1					
2	QSFS	.343**	1				
3	PF	.176**	.339**	1			
4	IADL	-.167**	-.216**	-.465**	1		
5	total score of WHOQOL-OLD	-.480**	-.272**	-.297**	.266**	1	
6	Living alone	.099*	.500**	.140**	.044	-.092	1

Note1: * $p < .05$; ** $p < .01$

Note2: QSFS (Questionnaire to define social frailty status); PF(physical frailty); IADL(Instrumental activities of daily living).

Note3: The references of physical frailty criteria given by Taiwanese studies are as follows (Chen, et al., 2014; Lin, et al., 2011; Liu, et al., 2017): (1) Weight loss was defined as unintentional reduction in 5% body weight or 3 kg over a 1-year period; (2) Poor grip strength is defined as less than 26 kg for men and less than 18 kg for women; (3) Poor endurance and energy were measured by self-reported exhaustion, identified by two questions from the “Center for Epidemiological Studies-Depression scale, CES-D”; (4) Slow walking speed was assessed for participants whose walking speed of 15 feet was over 0.8 sec/ meter or in the slowest 20% of the sample; (5) Physical activity is measured by “The International Physical Activity Questionnaire, IPAQ”. The lowest 20% of physical activity in our study sample was identified as low physical activity level. Participants who met none of these criteria were considered to be nonfrail, while those who met one or two criteria were defined as prefrail. Those who met three or more criteria were considered as being frail.

L. K. Chen et al., Sarcopenia in Asia: consensus report of the Asian Working Group for Sarcopenia. Journal of the American Medical Directors Association 15, 95-101 (2014).

C. C. Lin, et al., Reduced health-related quality of life in elders with frailty: A cross-sectional study of community-dwelling elders in Taiwan. PloS one, 6 (2011).

L. K. Liu, et al., Subtypes of physical frailty: Latent class analysis and associations with clinical characteristics and outcomes. *Scientific reports*, 7, 46417 (2017).

Table S7: Analysis of incremental validity (N=446; Dependent Variable=WHOOQOL)

	Model 1			Model 2			Model 3			Model 4		
independent variable	β	t	p-value	β	t	p-value	β	t	p-value	β	t	p-value
QSFS	-.273	-5.972	.000	-.123	-2.784	.006	-.059	-1.301	.194	-.054	-1.206	.228
SF-16				-.439	-9.976	.000	-.425	-9.868	.000	-.417	-9.694	.000
PF							-.202	-4.701	.000	-.158	-3.338	.001
IADL										.102	2.233	.026
R2	0.075			0.245			0.281			0.289		
Adj R2	0.072			0.241			0.276			0.282		

Table S8: Analysis of incremental validity (N=446; Dependent Variable=WHOOQOL) Changing QSFS/SF 16 order of entry

	Model 1			Model 2			Model 3			Model 4		
independent variable	β	t	p-value	β	t	p-value	β	t	p-value	β	t	p-value
SF-16	-.481	-11.547	.000	-.439	-9.976	.000	-.425	-9.868	.000	-.417	-9.694	.000
QSFS				-.123	-2.784	.006	-.059	-1.301	.194	-.054	-1.206	.228
PF							-.202	-4.701	.000	-.158	-3.338	.001
IADL										.102	2.233	.026
R2	0.231			0.245			0.281			0.289		
Adj R2	0.23			0.241			0.276			0.282		
